# Study on cellular uptake of a hydrophobic near-infrared-absorbing diradical-platinum(II) complex solubilized by albumin using hyperspectral imaging, spectrophotometry, and spectrofluorimetry

**DOI:** 10.1007/s44211-024-00621-8

**Published:** 2024-06-19

**Authors:** Ryota Sawamura, Atsuko Masuya-Suzuki, Nobuhiko Iki

**Affiliations:** 1 Graduate School of Environmental Studies, Tohoku University, 6-6-07 Aramaki-Aoba, Aoba-Ku, Sendai, 980-8579 Japan; 2 Graduate School of Sciences and Technology for Innovation, Yamaguchi University, 1677-1 Yoshida, Yamaguchi, 753-8511 Japan

**Keywords:** Hyperspectral imaging, Spectrophotometry, Spectrofluorimetry, Near-infrared, Diradical-platinum complex

## Abstract

Owing to its biopenetrability and minimal invasiveness, near-infrared (NIR) light in the region between 700–1100 nm has attracted attention in cancer diagnosis and therapy. Our group previously reported that the hydrophobic diradical-platinum(II) complex PtL_2_ is a promising agent for cancer photothermal therapy (L = 3,5-dibromo-1,2-diiminobenzosemiquinonate radical). Because PtL_2_ does not fluoresce, its intercellular uptake of PtL_2_ cannot be observed with a fluorescence microscope. In this study, we clarified the uptake and intracellular behavior of PtL_2_ solubilized by bovine serum albumin (BSA) using hyperspectral imaging enabling spectrophotometric analysis of the image. The spectral changes in the obtained images indicated that the internalization of PtL_2_ was followed by crystallization of the complex during the long incubation period (> 4 h). Additionally, the binding constant *K*_b_ = 5.91 × 10^4^ M^–1^ could be estimated upon fluorescence quenching analysis of BSA upon binding of PtL_2_; *K*_b_ is two orders of magnitude smaller than that of albumin–common drugs. Considering the small *K*_b_ and low solubility of PtL_2_ in water, we ultimately proposed the internalization path and fate of PtL_2_ in the cell: release of PtL_2_ from BSA near cellular membranes and subsequent cellular uptake via membrane permeation followed by saturation, resulting in crystallization.

**Graphical abstract:**

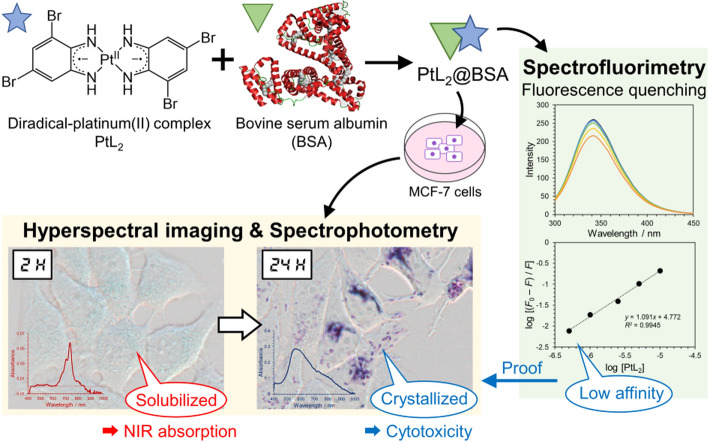

**Supplementary Information:**

The online version contains supplementary material available at 10.1007/s44211-024-00621-8.

## Introduction

In the near-infrared (NIR) region of 700–1100 nm, since biological components (water, lipid, hemoglobin, etc.) do not show strong absorption, NIR light can deeply penetrate biological tissues [1]. Moreover, the photon energy of NIR light is lower than those of X-rays and ultraviolet light, resulting in minimal damage to healthy tissues. These attractive features of NIR light have encouraged research into its applications in cancer diagnosis and phototherapy. Various types of NIR-absorbing materials have been developed as contrast and/or therapeutic agents. For example, fluorescent materials can be used for NIR fluorescence imaging [2, 3]. Non-fluorescent substances that generate heat via a nonradiative decay process or surface plasmon resonance can be used for photoacoustic imaging (PAI) and photothermal therapy (PTT) [4, 5]. Additionally, NIR-emissive materials with high luminescence quantum yields provide high signal-to-noise ratio images. Substances with nearly 100% photothermal conversion efficiency may enable PAI and PTT with fewer doses and/or lower irradiation powers. The internalization and intracellular behavior of NIR-absorbing substances have been studied using various techniques to enhance the performance of imaging and therapy using NIR light. For example, the intracellular localization of nanosized materials has been studied using electron microscopy or fluorescence microscopy after conjugation with fluorophores [6, 7]. The fluorescence of NIR organic dyes can be used directly to analyze subcellular localization [8, 9]. However, it is difficult for neither electron nor fluorescence microscopes to observe the molecular-sized and non-fluorescent NIR-absorbing materials. Conjugation of such materials with a fluorophore may allow microscopic observation but would change the physicochemical and biochemical characteristics of the original material. A new technique is required to enable the observation of cellular uptake and subsequent events of non-fluorescent NIR-absorbing molecules.

**Fig. 1:**
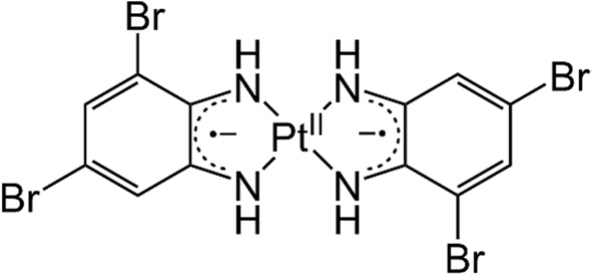
Chemical structure of the hydrophobic complex *trans*-PtL_2_

Diradical-platinum(II) complexes consisting of a Pt^II^ ion and two *o*-diiminobenzosemiquinonato radical ligands show an intense absorption band in the region of 700–800 nm derived from ligand-to-ligand charge transfer (LLCT) with molar absorption coefficients (*ε*) of 10^4^–10^5^ M^–1^ cm^–1^ [10]. The *ε* values are almost of the same order as those of cyanine dyes [11]. Our group has studied these complexes for their application as novel NIR-absorbing probes in response to pH or hydrophobic environments [12–14]. In addition, these complexes do not emit fluorescence, implying that they can efficiently convert the absorbed NIR light energy into heat. Recently, we discovered that the hydrophobic complex PtL_2_ that comprises bromo groups (L = 3,5-dibromo-1,2-diiminobenzosemiquinonate radical, Fig. 1) exhibited NIR absorption in cancer cells and killed cells by photothermal conversion, indicating that PtL_2_ can be used as a PTT agent [15]. Moreover, PtL_2_ has been used as a PAI probe for cancer cells [16]. However, the cytotoxic concentration (CC50) of PtL_2_ is as low as that of cisplatin, a typical anti-cancer drug [15, 17]. Because such intrinsic cytotoxicity may cause unexpected damage to healthy tissues, as in the case of conventional anti-cancer drugs, PtL_2_ should be selectively delivered to cancer tissues. More importantly, the cellular uptake, intracellular behavior, and biomolecular interactions of PtL_2_ should be elucidated from the viewpoint of drug design.

In this study, we investigated the uptake of non-fluorescent NIR-absorbing PtL_2_ solubilized by bovine serum albumin (BSA) (denoted as PtL_2_@BSA) in cancer cells using various spectroscopic methods: (1) Spectrophotometry including hyperspectral imaging of PtL_2_ in the cells providing spectral information in each pixel [18], clarifying the location and form of PtL_2_. (2) Fluorescence quenching analysis of the binding affinity of PtL_2_ for BSA. Based on these experiments, we propose a cellular uptake mechanism and the chemical form of PtL_2_ in cells.

## Experimental

### Reagents

Potassium tetrachloroplatinate(II) (K_2_PtCl_4_), hydrochloric acid (HCl), nitric acid (HNO_3_), *N*,*N*-dimethylformamide (DMF), and dimethyl sulfoxide (DMSO) were purchased from Kanto Chemical Co., Inc. (Tokyo, Japan). 3,5-Dibromo-1,2-phenylenediamine monohydrochloride (H_2_L·HCl) was supplied from Tokyo Chemical Industry Co., Ltd. (Tokyo, Japan). Hydrogen peroxide (H_2_O_2_) and phosphate-buffered saline (PBS, in powder form) were purchased from FUJIFILM Wako Pure Chemical Corporation (Osaka, Japan). BSA was purchased from Sigma-Aldrich. Reagents for cell culture, RPMI 1640 medium, fetal bovine serum (FBS), penicillin/streptomycin (PS), and trypsin/ethylenediaminetetraacetic acid (trypsin/EDTA)) were purchased from ThermoFisher Scientific. All reagents and materials were used without further purification.

### Synthesis of PtL_2_

The complex PtL_2_ was synthesized based on the reported method [19]. H_2_L·HCl (0.046 g, 0.150 mmol) was added to the HCl solution (0.01 M, 9 mL) of K_2_PtCl_4_ (0.031 g, 0.075 mmol) and then heated at 70 °C with stirring for 24 h under argon atmosphere. After being added water/DMF 1:1 (v/v, 18 mL), the reaction mixture was filtered to remove insoluble substances. The obtained filtrate was heated with shielding light at 50 °C for 2 days in the presence of air. The blue-violet precipitates were collected by filtration, washed with water/DMF 1:2, 1:1 (v/v), and water, and finally dried in vacuo (0.023 g, yield 43%). The complex PtL_2_ was used as the mixture of *cis* and *trans* isomers. ^1^H NMR (400 MHz, DMSO-*d*_6_): *δ* 7.21/7.22 (dd, *J*_1_ = 1.60/2.00 Hz, *J*_2_ = 4.40/4.80 Hz, 1H, Ar*H*), 7.42 (br s, 1H, Ar*H*). C_12_H_8_N_4_Br_4_Pt (722.92): calcd. C 19.94, H 1.12, N 7.75; found C 20.02, H 1.25, N 7.66.

### Preparation of PtL_2_@BSA in phosphate-buffered saline (PBS)

The BSA solution in PBS (1 mM, 2 mL) and PtL_2_ in DMSO (1 mM) were mixed and the volume was filled up to 20 mL by PBS. The mixture solution was warmed at 37 °C overnight and then filtered using a hydrophilic polytetrafluoroethylene (PTFE) membrane to remove precipitates. The collected filtrate was put in a centrifugal concentration tube Vivaspin Turbo 15 (polyethersulfone (PES) membrane, MWCO 30 kDa) and centrifuged at 3260×*g* for 24.5 min. The obtained concentrate was resuspended in PBS (15 mL) and centrifuged again at the same condition. After that, the concentrate was collected as the PBS solution of PtL_2_@BSA.

The concentration of PtL_2_ was determined by inductively coupled plasma atomic emission spectroscopy (ICP-AES) using Thermo iCAP 6500 Spectrometer. The PtL_2_ solution solubilized in PBS (100 μL) was heated with concentrated HNO_3_ (1 mL) and 30% H_2_O_2_ (1 mL) at 95 °C overnight and completely dried. The residue was dissolved in 0.1 M HNO_3_ (20 mL) to afford the sample solution.

### Cell culture

Human breast cancer cell line MCF-7 cells were obtained from Cell Resource Center for Biomedical Research, Institute of Development, Aging and Cancer, Tohoku University (Miyagi, Japan). The cells were cultured in RPMI 1640 medium supplemented with 10% FBS and 1% PS at 37 °C under the humidified atmosphere of 5% CO_2_. Cells were passed by splitting with trypsin/EDTA when they were 70–80% confluent.

### Measurement of the quantity of Pt in cell suspension

MCF-7 cells (2 × 10^4^ cells cm^–2^) were preincubated in a φ35 mm culture dish at 37 °C for 24 h. The cells were then incubated in the culture medium supplemented with PtL_2_@BSA ([PtL_2_] = 20 μM) at 37 °C for 2 h. After rinsing with PBS two times, the cells were peeled off from the dishes and suspended in PBS (500 µL). Concentrated HNO_3_ (1 mL) and 30% H_2_O_2_ (1 mL) were added to the cell suspension and heated at 95 °C overnight and completely dried. The residue was dissolved in 0.1 M HNO_3_ (20 mL) to afford the sample solution. The measurement was performed by Thermo iCAP 6500 Spectrometer. The amount of Pt was calculated by multiplying the obtained Pt concentration by the suspension volume.

### Hyperspectral imaging of MCF-7 cells containing the complex

MCF-7 cells (2 × 10^4^ cells cm^–2^) were preincubated in a φ35 mm culture dish at 37 °C for 24 h. The cells were then incubated in the culture medium supplemented with PtL_2_@BSA ([PtL_2_] = 20 μM) at 37 °C for 2 h. After rinsing with PBS two times, the cells were observed using the microscope with the hyperspectral camera.

The hyperspectral camera NH-1TIK (EBA JAPAN Co., Ltd., Tokyo, Japan) can take images (640 × 480 pixels) having spectral information (400–1000 nm, wavelength resolution: 5 nm, 121 wavelength points). Color images were constructed by assigning three sub-images (image at each wavelength) at 480, 545 nm, and 700 nm, to blue, green, and red, respectively. Absorption spectra of the region of interest (ROI) were obtained by plotting the absorbance (*A*_*λ*_) in ROI against the wavelength *λ*. The *A*_*λ*_ value was calculated by the following equation based on the relationship between the absorbance and transmittance,

1 $$\begin{array}{c}{A}_{\lambda }=-\text{log}\frac{I\left(\lambda \right)}{{I}_{\text{BG}}\left(\lambda \right)}.\end{array}$$

*I*(*λ*) and *I*_BG_(*λ*) represent the average incident light intensity in pixels of ROI when the cells were present or absent, respectively.

### Absorption and fluorescence spectra measurements

Absorption spectra were measured using a Shimadzu UV-1800 UV-Vis spectrometer attaching temperature-controllable cell holders TCC-240A (Shimadzu). Samples were transferred to two-sided transparent quartz cuvettes (light path length: 10 mm, width: 10 mm for solutions, or 2 mm for cell suspensions) and measured at 37 °C. Fluorescence spectra were measured by setting a four-sided transparent quartz cuvette containing the sample on a HITACHI F-7000 fluorescence spectrophotometer. The excitation wavelength was set to 280 nm. The scan speed was 240 nm min^–1^. The widths of excitation/emission slits were set to 5.0/5.0 nm. The photomultiplier tube voltage was 400 V.

## Results and discussion

### Time dependence of the internalization of PtL_2_ into cells

**Fig. 2:**
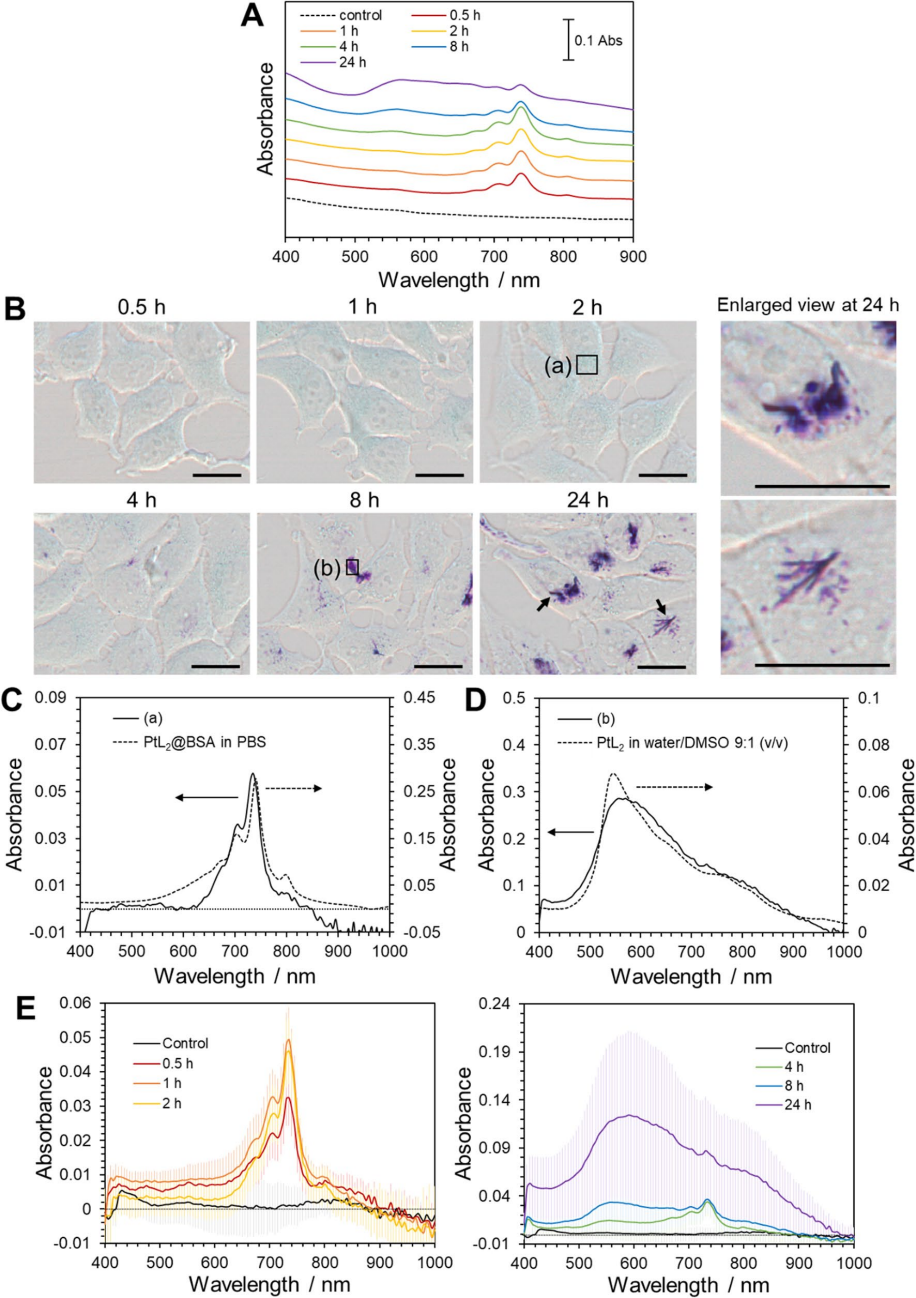
Absorption spectra of the suspensions of human breast cancer MCF-7 cells incubated with PtL_2_@BSA ([PtL_2_] = 20 µM) for different periods (**A**). Color images of MCF-7 cells incubated with PtL_2_@BSA ([PtL_2_] = 20 µM) for different periods (**B**). Scale bars represent 10 µm. The enlarged images at 24 h correspond to the arrows in the overall one. Absorption spectra of (a) in the image at 2 h (solid line, left axis) and PtL_2_@BSA in PBS (dashed line, right axis) (**C**). Absorption spectra of (b) in the image at 8 h (solid line, left axis) and PtL_2_ in water/DMSO 9:1 (v/v, dashed line, right axis) (**D**). The average absorption spectra of the region near nuclei for 28 cells incubated with PtL_2_@BSA at 37 °C for 0.5–2 h (left) and 4–24 h (right) (**E**). The ‘Control’ cells were incubated without PtL_2_@BSA for 2 h. Error bars represent the standard deviation. *n* = 28

MCF-7 cells were incubated with PtL_2_@BSA for different time periods and cell suspensions were prepared. As shown in Fig. 2A, the absorption spectra of the cell suspensions comprised NIR absorption peaks at 739 nm for up to 4 h; the peaks are assignable to the LLCT band of PtL_2_. PtL_2_ exhibits NIR absorption at 738 nm in egg lecithin (phosphatidylcholine) multilamellar vesicles mimicking biological membranes [12], thus implying that the complex localizes to the cellular membranes of MCF-7 cells. After 4 h, a broad absorption peak emerged at approximately 550 nm and the intensity of the peak at 739 nm gradually decreased. A broad absorption band at ~ 550 nm is also observed for PtL_2_ in water-rich water/DMSO mixed solvents, which is attributed to the suspension of insoluble PtL_2_ particles, as evidenced by the Tyndall effect during irradiation with a green laser pointer (Figure S1). Therefore, the broad absorption at approximately 550 nm in the cell suspension (Fig. 2A) may also represent insoluble PtL_2_.

Next, the PtL_2_-introduced cells were observed using a microscope equipped with a hyperspectral imaging camera (Fig. 2B). Color images of these cells initially contained blue areas, and violet areas gradually emerged from 4 h onwards. The absorption spectrum of the black-framed region (a) in the color image at 2 h comprised an absorption peak at 735 nm (solid line in Fig. 2C). A blue shift in the peak wavelength compared with that of PtL_2_@BSA in PBS (740 nm, dashed line in Fig. 2C) suggests that PtL_2_ is present in an environment different from that in BSA; the environment could be that of a cellular membrane. The black framed region (b) in the color image shows a broad absorption peak at 8 h at approximately 550 nm (solid line in Fig. 2D). This spectrum resembles that of PtL_2_ in a water-rich water/DMSO mixed solvent (9:1, v/v, dashed line in Fig. 2D, and Figure S1). Furthermore, needle-like substances were observed in the violet areas (Fig. 2B, the enlarged views represent the arrowed area in the image at 24 h), indicating crystallization of PtL_2_. Because the complex showing NIR absorption exists in the region surrounding the cell nuclei [15], we then measured the absorption spectra in the region near the nuclei of several cells (*n* = 28, Figure S2) and calculated the average spectra (Fig. 2E). The absorption peak at 735 nm was the largest at 2 h and decreased thereafter. By contrast, the broad absorption peak at 550–600 nm gradually increases in intensity. These spectral changes were similar to those observed in cell suspensions (Fig. 2A).

To investigate the temporal changes in the cellular uptake of PtL_2_, we measured the quantity of Pt in the cell suspension using ICP-AES. As the incubation time increases, the quantity of Pt increases almost linearly (Figure S3). Therefore, PtL_2_ was steadily internalized into the cells, and part of the complex gradually crystallized.

### Effect of temperature on the cellular uptake of PtL_2_

**Fig. 3:**
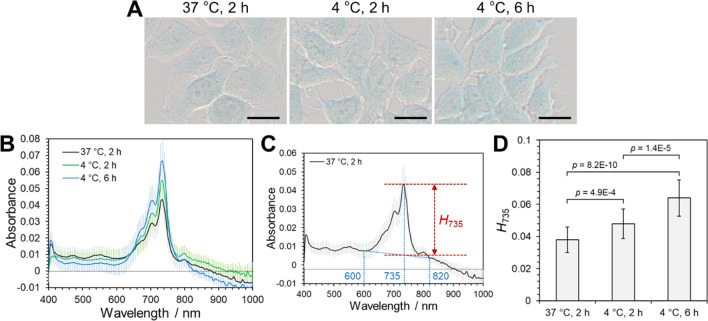
Color images of MCF-7 cells incubated with solubilized PtL_2_ ([PtL_2_] = 20 µM) at 37 or 4 °C for 2 or 6 h (**A**). Scale bars represent 10 µm. The average absorption spectra of blue-colored areas in the cells incubated with PtL_2_@BSA ([PtL_2_] = 20 µM) at 37 °C for 2 h (black line), at 4 °C for 2 h (green line), or at 4 °C for 6 h (blue line) (**B**). Calculation method of the peak height at 735 nm (*H*_735_) (**C**). The *H*_735_ values of three experimental conditions (**D**). Error bars represent the standard deviation. *n* = 20

If the cellular uptake of PtL_2_ is based on the endocytic pathways, incubation at low temperatures will inhibit or suppress its internalization. This is because the membrane fluidity decreases at lower temperatures than the phase transition temperature of phospholipids from the liquid–crystal to the gel phase [20]. We then incubated MCF-7 cells with PtL_2_@BSA at 4 °C. Absorption spectra of the cell suspensions at 4 °C at 2 and 6 h comprised an absorption band at 739 nm assignable to PtL_2_, whereas a band assignable to the crystallized PtL_2_ did not appear (Figure S4). Only blue areas were present in the cells under all three incubation conditions (Fig. 3A) in the color images captured using the hyperspectral imaging camera. The average absorption spectra obtained from the blue-colored areas of 20 cells (Figure S5) indicated the absorption peaks at 735 nm (Fig. 3B). For quantitative analysis of the uptake of PtL_2_, we defined the peak height at 735 nm (*H*_735_), as shown in Fig. 3C. The *H*_735_ values became larger by lowering the temperature and elongating the incubation time at 4 °C (Fig. 3D). Considering the intracellular quantity of Pt (Figure S3), the cellular uptake of PtL_2_ increased with incubation time instead of with increasing temperature. These results imply that the complex might not be internalized via energy-dependent endocytic pathways but permeates the membranes passively after being released from BSA. The reason why PtL_2_ did not crystallize by prolonged incubation at 4 °C might be that the amplitude of the thermal motion of PtL_2_ was decreased at low temperatures, and the complex remained inside the membranes of organelles and/or cytoplasmic membranes following the phase transition of phospholipids.

### Analysis of the binding affinity of PtL_2_ to BSA

**Fig. 4:**
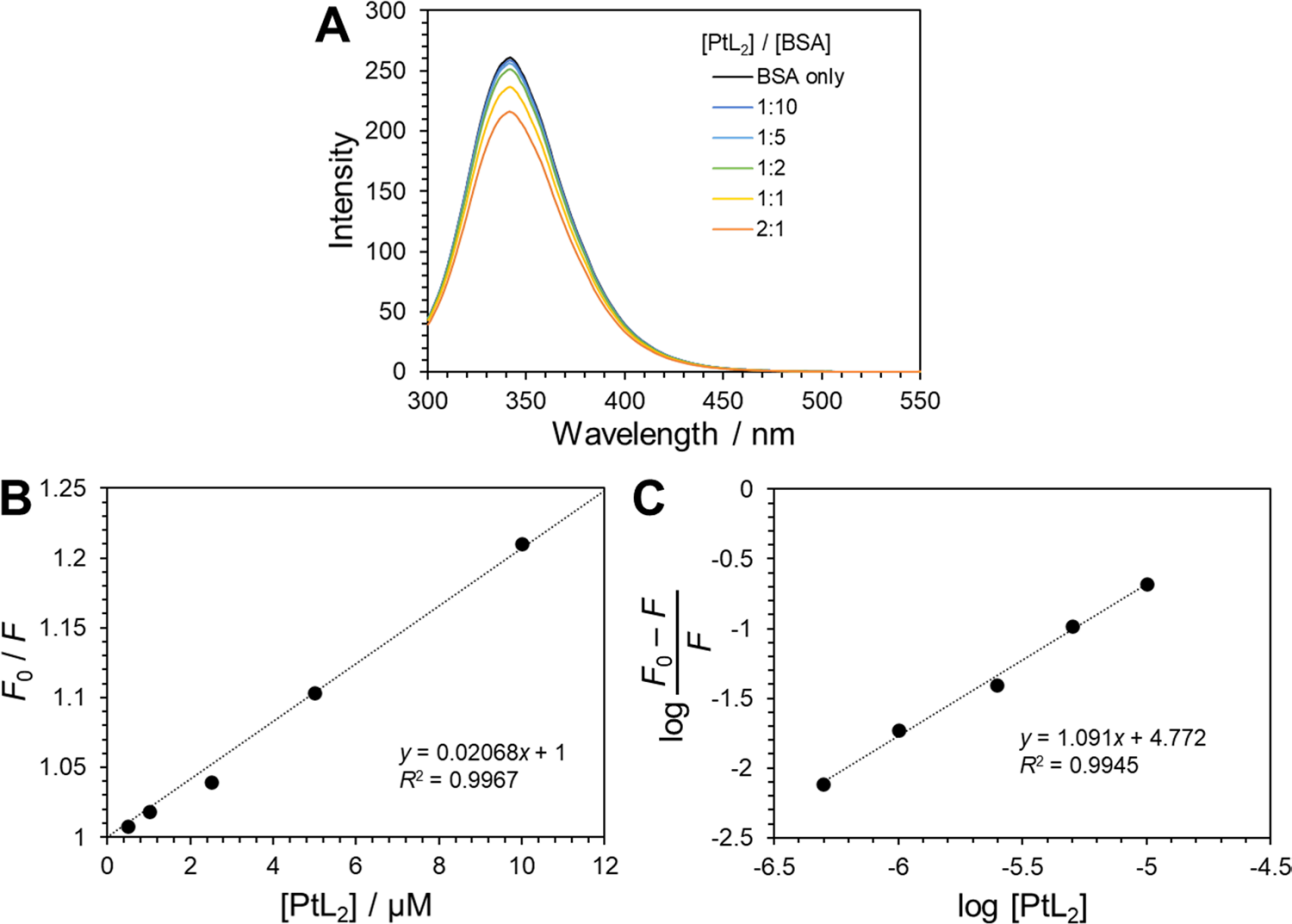
Fluorescence spectra of BSA mixed with PtL_2_ at different concentrations at 37 °C for 24 h (**A**). [PtL_2_] = 0, 0.5, 1.0, 2.5, 5.0, 10 µM; [BSA] = 5.0 µM. PBS/DMSO 199:1 (v/v). The excitation wavelength was 280 nm. Plot of the *F*_0_/*F* ratio versus the PtL_2_ concentration (**B**). Plot of $\text{log}\frac{{F}_{0}-F}{F}$ versus log[PtL_2_] (**C**)

To investigate the possibility that PtL_2_ is released from BSA during its internalization into cells, the binding affinity of PtL_2_ to BSA was investigated by fluorescence quenching analysis. Proteins emit intrinsic fluorescence from their aromatic amino acids. When a molecule acts as a quencher, the binding stoichiometry and constant of the molecule to the protein can be calculated from the change in fluorescence intensity [21]. We measured the fluorescence spectra of BSA in the presence of different concentrations of PtL_2_ (Fig. 4A). The intensity of the luminescence peak at 342 nm decreased with the addition of PtL_2_. Generally, two types of quenching occur, namely, static and dynamic. For the latter, Eq. (2) holds.

2 $$\begin{array}{c}\frac{{F}_{0}}{F}=1+{k}_{q}{\tau }_{0}\left[{\text{Q}}\right],\end{array}$$

where *F* and *F*_0_ [-] are the fluorescence intensities in the presence and absence of quenchers, respectively, *k*_*q*_ [M^–1^ s^–1^] is the quenching rate constant, *τ*_0_ [s] is the fluorescence lifetime in the absence of quenchers, and [Q] [M] is the concentration of quenchers [21]. Figure 4B shows the plot of the *F*_0_/*F* ratio versus the PtL_2_ concentration, and *k*_*q*_ was estimated to be 3.5 × 10^12^ M^–1^ s^–1^ while *τ*_0_ of native BSA is reported to be 6.0 ns [22]. The value of *k*_*q*_ is larger than the largest possible value for diffusion-controlled bimolecular quenching in aqueous solutions (1 × 10^10^ M^–1^ s^–1^) [21], indicating that BSA fluorescence is mainly quenched via the static mechanism in the presence of PtL_2_.

When *n* molecules of PtL_2_ are bound to one molecule of BSA, the binding constant *K*_b_ is represented as

3 $$\begin{array}{c}{K}_{b}=\frac{\left[{\text{Pt}}{\text{L}}_{2}\text{@}{\text{BSA}}\right]}{{\left[{\text{Pt}}{\text{L}}_{2}\right]}^{n}\left[{\text{BSA}}\right]}.\end{array}$$

Because PtL_2_@BSA is non-fluorescent, the [PtL_2_@BSA]/[BSA] ratio can be written using the fluorescence intensity *F* as follows:

4 $$\begin{array}{c}\frac{\left[{\text{Pt}}{\text{L}}_{2}\text{@}{\text{BSA}}\right]}{\left[{\text{BSA}}\right]}=\frac{{F}_{0}-F}{F}.\end{array}$$

From Eq. (3),

5 $$\begin{array}{c}\frac{{F}_{0}-F}{F}={K}_{b}{\left[{\text{Pt}}{\text{L}}_{2}\right]}^{n}.\end{array}$$

Taking the logarithm of both sides,

6 $$\begin{array}{c}\text{log}\frac{{F}_{0}-F}{F}=\text{log}{K}_{b}+n\text{log}\left[{\text{Pt}}{\text{L}}_{2}\right].\end{array}$$

The binding constant *K*_b_ and number of binding sites *n* can be determined from the intercept and slope of the plot of $\text{log}\frac{{F}_{0}-F}{F}$ versus log[PtL_2_], respectively. Thus, *K*_b_ and *n* are 5.91 × 10^4^ M^–1^ and 1.09, as determined from the plot of $\text{log}\frac{{F}_{0}-F}{F}$ versus log[PtL_2_] (Fig. 4C) The *K*_b_ value of PtL_2_ with BSA is nearly two orders of magnitude smaller than that with common drugs such as human serum albumin (HSA). For example, *K*_b_ values of the anti-inflammatory drug ibuprofen and anti-cancer drug paclitaxel are 10^5^–10^6^ M^–1^ [23] and 10^6^ M^–1^ [24], respectively. Although the binding ratio of PtL_2_ to BSA was 1:1, 50 times more BSA was required for the absorption spectrum, showing the LLCT band of PtL_2_ almost completely (Figure S6). These results imply that the stability of PtL_2_@BSA complex is low and readily releases PtL_2_ without the presence of a large quantity of BSA.

### Proposed paths of cellular uptake and intracellular behavior of PtL_2_

**Scheme 1:**
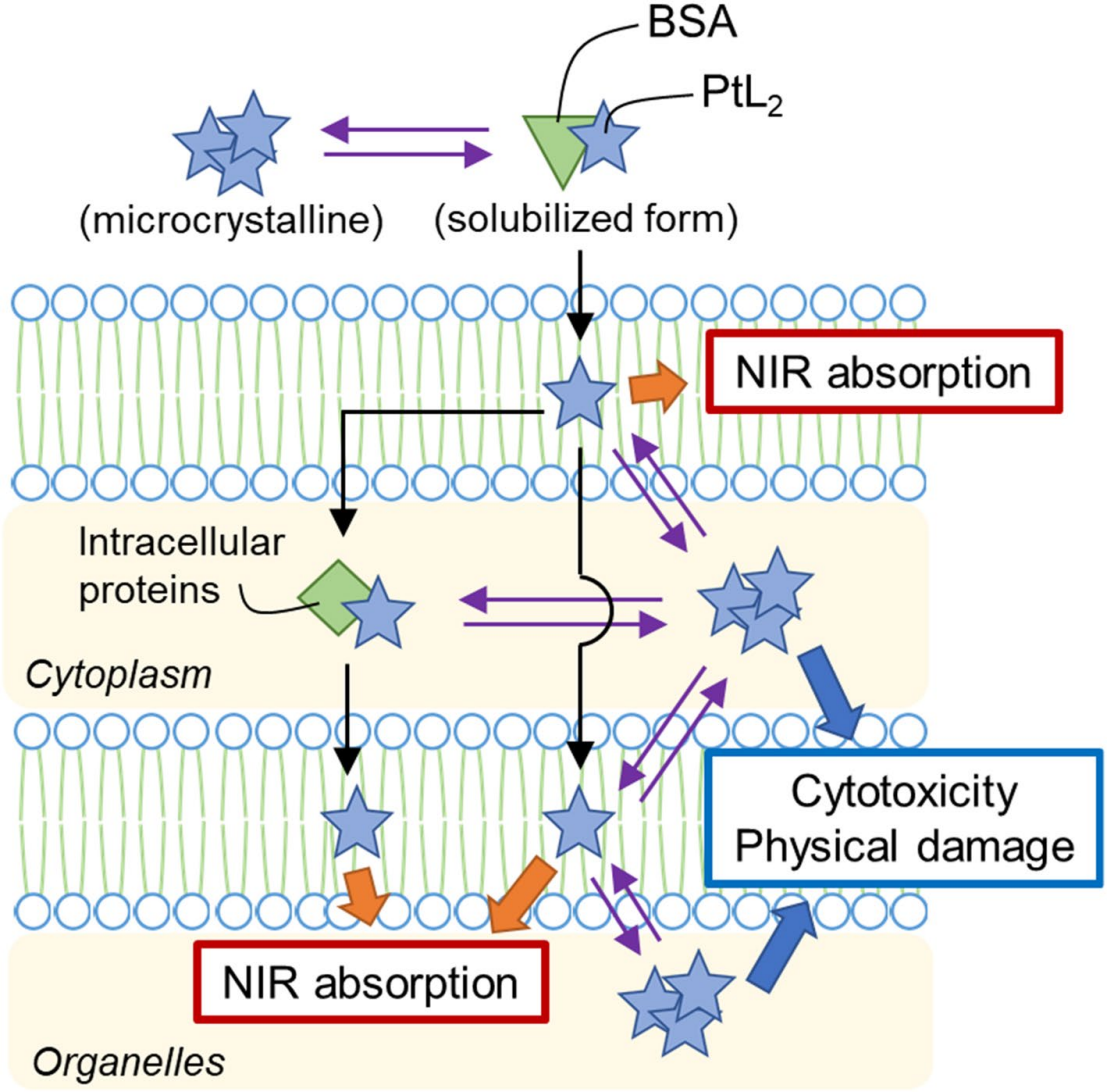
Proposed pathway of internalization and crystallization of PtL_2_ into cells

Based on the above discussion, the internalization and crystallization pathway of PtL_2_ is proposed (Scheme 1). The complex that exists in aqueous solutions containing BSA will be balanced in two equilibria, the association equilibrium with BSA and the solubility equilibrium of PtL_2_ itself. Since the complex is quite insoluble in water, it can be predicted that the overall equilibria will be easily shifted to the solid state without the presence of sufficient amount of BSA. The complex released from BSA near the cellular surface may be incorporated in the hydrophobic inner side of the membrane sooner than the solidification outside the cells. This process is promoted by the free energy change during the translocation of hydrophobic compounds from the aqueous phase into the lipid bilayers [25]. Some complexes permeate the cytoplasmic membrane and enter the cytoplasm. They can move through the cytoplasm in free or protein-bound (such as albumin) forms and enter organelle membranes. As the intracellular quantity of Pt increases with increasing incubation time (Figure S3), the hydrophobic areas where PtL_2_ can enter will eventually become saturated. The excess complexes would crystallize in the cells based on the solubility equilibrium.

The grown crystals might cause cytotoxicity eventually. The cells were alive under the incubation conditions ([PtL_2_] = 20 µM, 37 °C, 2 h) in which PtL_2_ did not crystallize in the cells [15]. By contrast, the cell viability drastically decreased by the 24 h exposure at the same concentration and incubation temperature [15]. At this time, we consider that the grown crystals shown in Fig. 2B could have damaged organelles physically, resulting in cytotoxicity. On the other hand, in the former case, it may be that PtL_2_ in the solubilized state did not show cytotoxicity simply due to the short exposure. Furthermore, no NIR absorption derived from PtL_2_ was observed in the nuclei [15]. We should note that the hyperspectral imaging camera used in this study cannot observe the substances not showing the characteristic absorption band in 400–1000 nm. Therefore, the possibility cannot be excluded that the degradation products of PtL_2_ intercalate with DNA as in the case of cisplatin [26]. A more detailed discussion on cytotoxicity of PtL_2_ is the subject of future research.

## Conclusion

We investigated the cellular uptake and intracellular forms of the hydrophobic diradical-platinum(II) complex PtL_2_ solubilized by BSA using hyperspectral imaging and absorption/fluorescence spectrometry. Observation of MCF-7 cells by the hyperspectral imaging camera clarified that PtL_2_ initially showed NIR absorption within the cells, but gradually crystallized after 4–24 h, leading to decreased NIR absorption. During this period, the quantity of intracellular PtL_2_ increased with time during incubation. By contrast, the complex was introduced into the cells at 4 °C without crystallization, suggesting uptake through permeation. Furthermore, the binding constant *K*_b_ of PtL_2_ to BSA was smaller than that of typical drugs. Based on these results, we propose the following internalization mechanism and fate: (1) PtL_2_ released from BSA near the cellular surface is internalized into cells by membrane permeation. (2) The quantity of PtL_2_ inside the lipid bilayer reached saturation. (3) Excess PtL_2_ crystallizes in the cell. For safe delivery of PtL_2_, carriers that can stably encapsulate the complexes are required. Additionally, developing complexes with a proper hydrophilic and lipophilic balance to maintain solubility and binding to the membrane by changing the substituent groups of the ligands is an alternative solution.

## Supplementary Information

Below is the link to the electronic supplementary material.

Supplementary file1 (DOCX 5637 KB)

## Data Availability

The datasets generated and/or analyzed during the current study are available from the corresponding authors on reasonable request.
