# Supplementary material for: Study on cellular uptake of a hydrophobic near-infrared-absorbing diradical-platinum(II) complex solubilized by albumin using hyperspectral imaging, spectrophotometry, and spectrofluorimetry
Source: Anal Sci. 2024 Jun 19;40(10):1857–65. doi: 10.1007/s44211-024-00621-8 (PMC11422251; doi:10.1007/s44211-024-00621-8)
Supplement: Supplementary file 1 — Supplementary file1 (DOCX 5637 KB) [file 44211_2024_621_MOESM1_ESM.docx]

**Supplementary information**

**Study on cellular uptake of a hydrophobic near-infrared-absorbing diradical-platinum(II) complex solubilized by albumin using hyperspectral imaging, spectrophotometry, and spectrofluorimetry**

Ryota Sawamura,^1,^* Atsuko Masuya-Suzuki,^2^ and Nobuhiko Iki^1,†^

^1^ *Graduate School of Environmental Studies, Tohoku University, 6-6-07 Aramaki-Aoba, Aoba-ku, Sendai 980-8579, Japan*

^2^ *Graduate School of Sciences and Technology for Innovation, Yamaguchi University, 1677-1 Yoshida, Yamaguchi 753-8511, Japan*

* [sawamura@tohoku.ac.jp](mailto:sawamura@tohoku.ac.jp)

† [iki@tohoku.ac.jp](mailto:iki@tohoku.ac.jp)

Contents

p. 2 Absorption spectra and photographs of PtL_2_ in different volume ratios of water/DMSO mixture solvents (**Figure S1**)

p. 3 The region near nuclei used for calculating average absorption spectra of MCF-7 cells incubated with PtL_2_@BSA for different periods (**Figure S2**)

p. 4 The relationship between the amount of Pt in cell suspensions and the incubation time (**Figure S3**)

p. 5 Absorption spectra of the suspended cells incubated with PtL_2_@BSA in different temperatures and incubation times (**Figure S4**)

p. 6 The region of blue-colored areas used for calculating average absorption spectra of MCF-7 cells incubated with PtL_2_@BSA in different temperatures and incubation times (**Figure S5**)

p. 7 Absorption spectra of PtL_2_ mixed with different molar ratios of BSA (**Figure S6**)


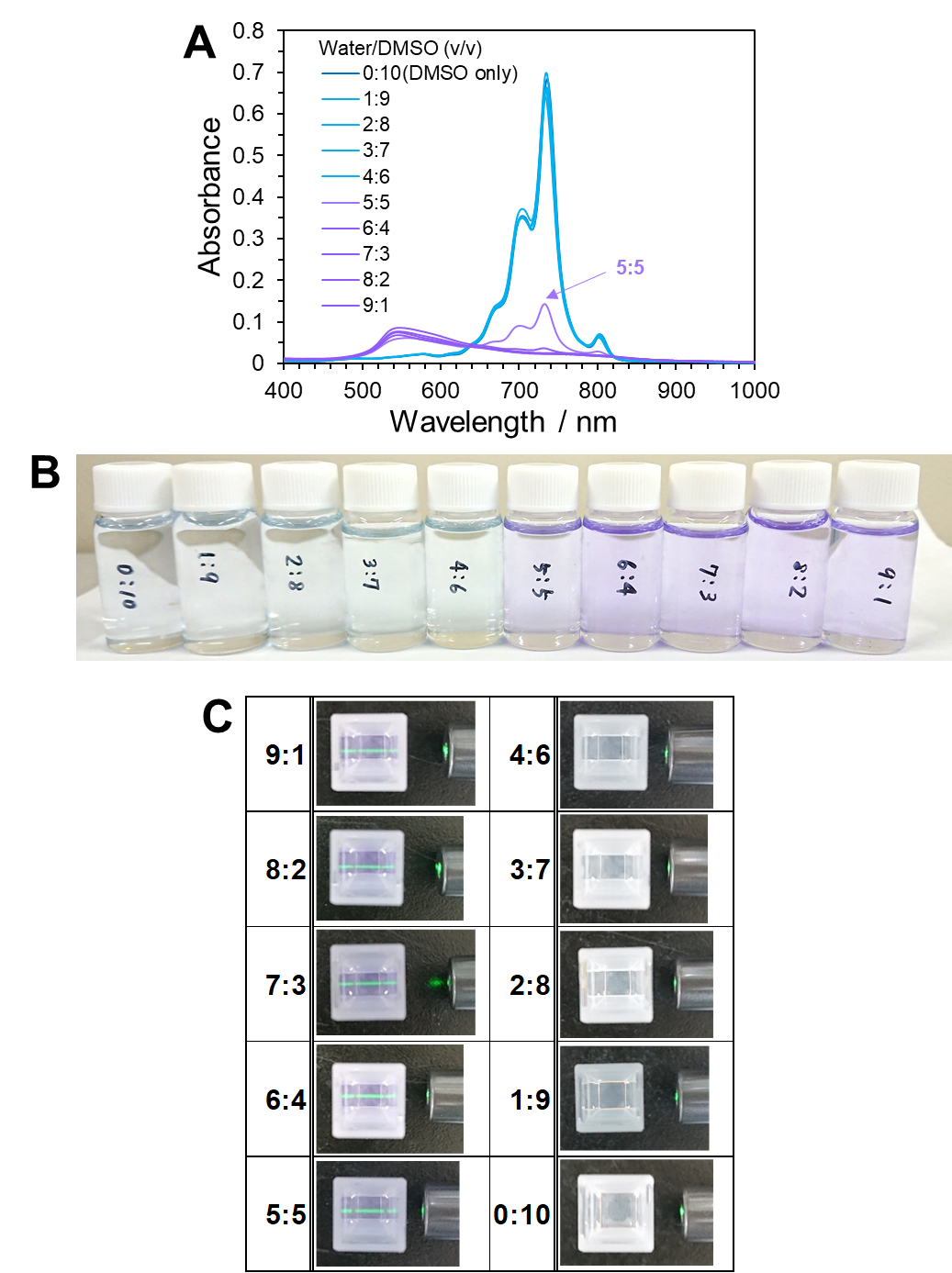


**Figure S1** Absorption spectra of PtL_2_ in different volume ratios of water/DMSO mixture solvents (**A**). [PtL_2_] = 5.0 µM. Water/DMSO 0:10 (DMSO only), 1:9, 2:8, 3:7, 4:6, 5:5, 6:4, 7:3, 8:2, and 9:1. The photograph of all samples (**B**). Photographs of the top view of the cells containing samples irradiated by a green laser pointer (**C**).


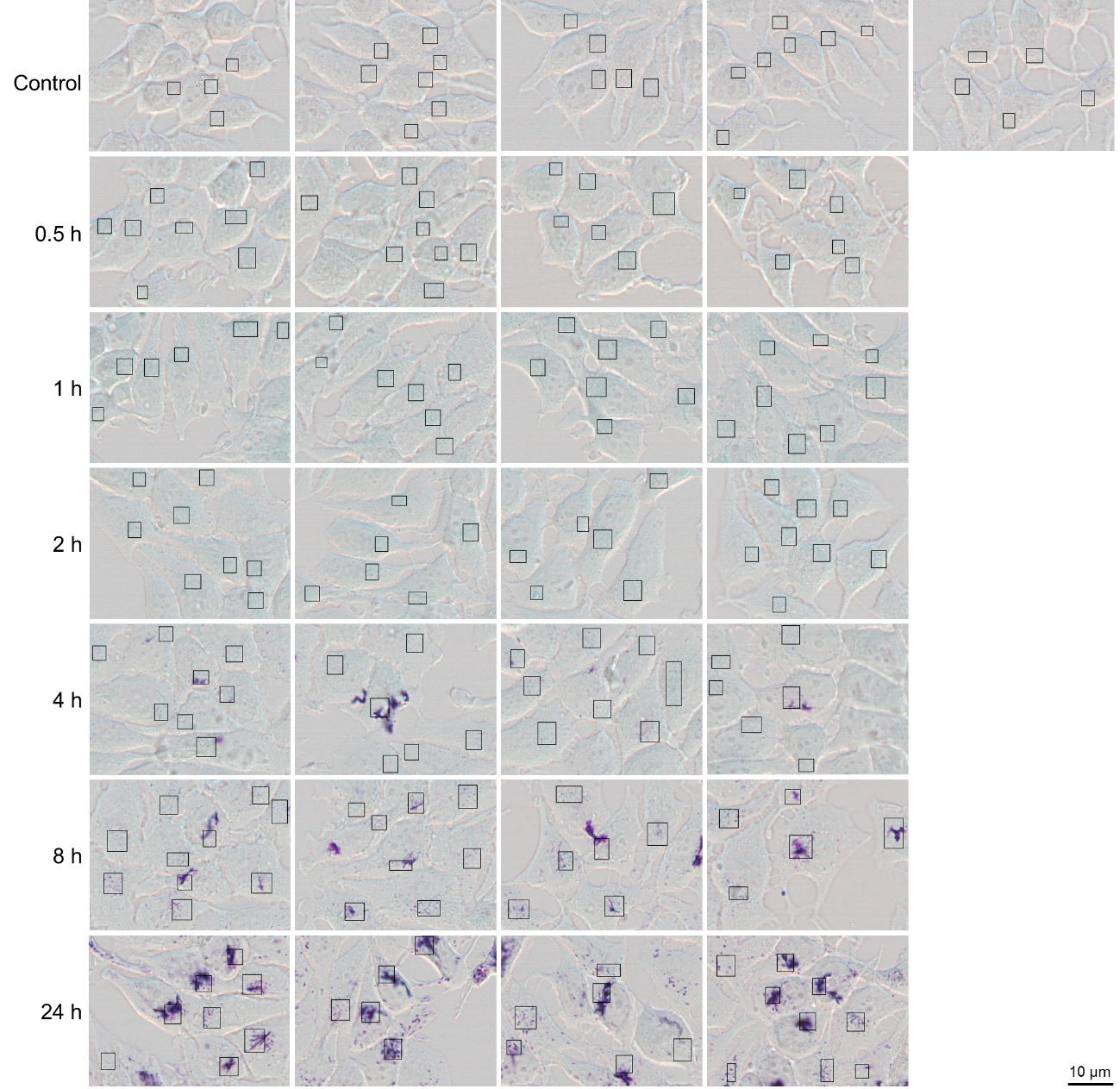


**Figure S2** The region near nuclei used for calculating average absorption spectra (**Figure 2E**). These cells were incubated with PtL_2_@BSA at 37 °C for 0.5–24 h. The ‘Control’ cells were incubated without PtL_2_@BSA for 2 h. The scale bar represents 10 µm. *n* = 28.


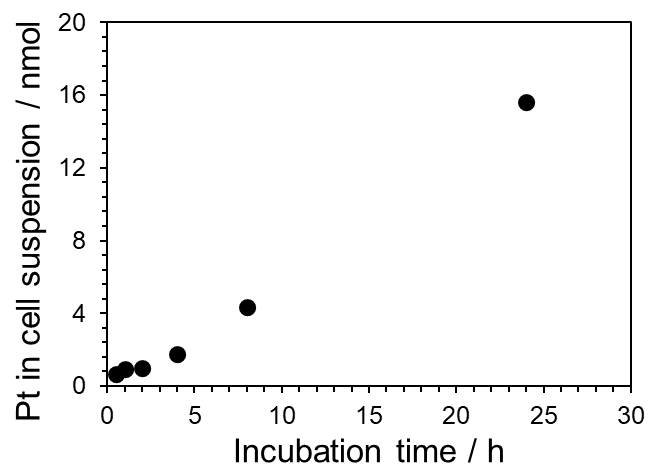


**Figure S3** Time courses of the quantity of Pt in the cell suspension (500 µL). The cells were incubated with solubilized PtL_2_ ([PtL_2_] = 20 µM) for different periods.


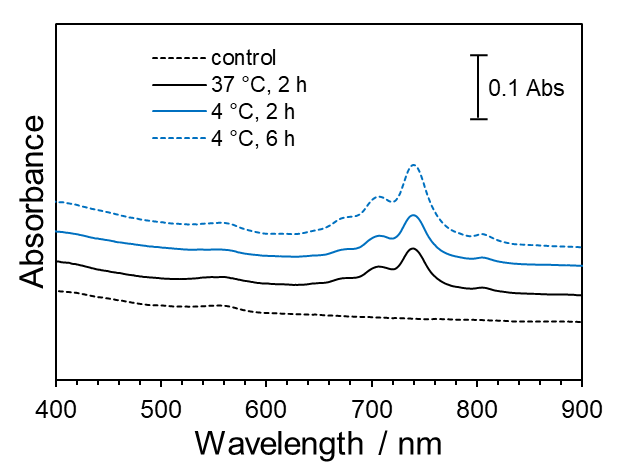


**Figure S4** Absorption spectra of the suspended cells incubated with PtL_2_@BSA ([PtL_2_] = 20 µM) at 37 °C for 2 h (black solid line), at 4 °C for 2 h (blue solid line), and 6 h (blue dashed line). The ‘control’ cells were incubated without PtL_2_.


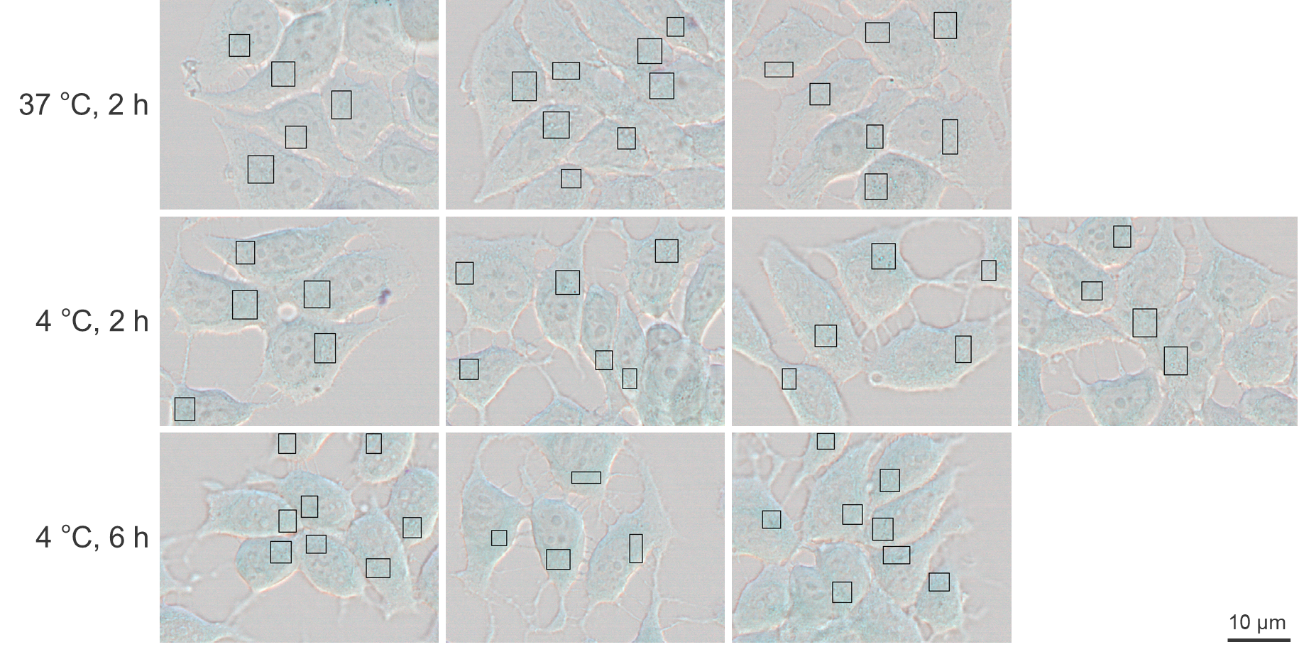


**Figure S5** The region of blue-colored areas used for calculating average absorption spectra (**Figure 3B**). These cells were incubated with PtL_2_@BSA ([PtL_2_] = 20 µM) at 37 °C for 2 h, at 4 °C for 2 h, and 6 h. The scale bar represents 10 µm. *n* = 20.


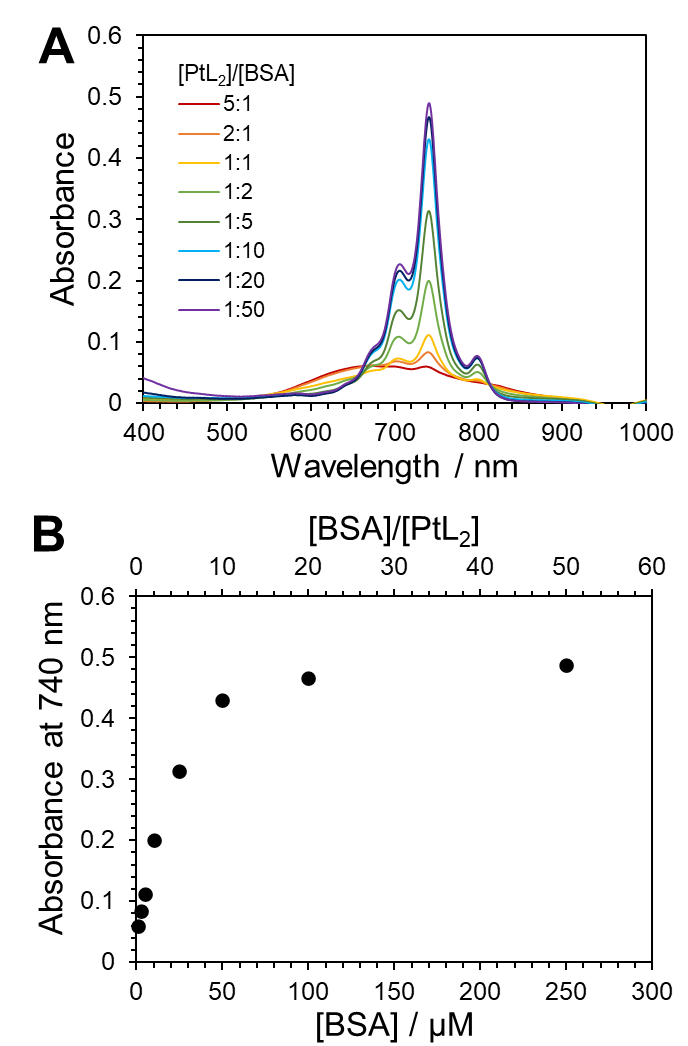


**Figure S6** The absorption spectra of PtL_2_/BSA mixture solutions at different PtL_2_/BSA molar ratios (**A**). [PtL_2_] = 5.0 µM. [BSA] = 1.0, 2.5, 5.0, 10, 25, 50, 100, 250 µM. PBS/DMSO 199:1 (v/v). The mixture solutions were warmed at 37 °C for 24 h before measurement. The relationship between the absorbance at 740 nm and BSA concentration (**B**).
